# Juvenile socio‐sexual experience determines lifetime sperm expenditure and adult survival in a polygamous moth, *Ephestia kuehniella*

**DOI:** 10.1111/1744-7917.13088

**Published:** 2022-06-22

**Authors:** Junyan Liu, Xiong Z. He, Xia‐Lin Zheng, Yujing Zhang, Qiao Wang

**Affiliations:** ^1^ School of Agriculture and Environment Massey University Palmerston North New Zealand; ^2^ Guangxi Key Laboratory of Agric‐Environment and Agric‐Products Safety National Demonstration Center for Experimental Plant Science Education, College of Agriculture, Guangxi University Nanning China

**Keywords:** juvenile experience, Lepidoptera, mating frequency, sperm allocation, sperm production, sperm ratio

## Abstract

Male animals often adjust their sperm investment in response to sperm competition environment. To date, only a few studies have investigated how juvenile sociosexual settings affect sperm production before adulthood and sperm allocation during the first mating. Yet, it is unclear whether juvenile sociosexual experience (1) determines lifetime sperm production and allocation in any animal species; (2) alters the eupyrene : apyrene sperm ratio in lifetime ejaculates of any lepidopteran insects, and (3) influences lifetime ejaculation patterns, number of matings and adult longevity. Here we used a polygamous moth, *Ephestia kuehniella*, to address these questions. Upon male adult emergence from juveniles reared at different density and sex ratio, we paired each male with a virgin female daily until his death. We dissected each mated female to count the sperm transferred and recorded male longevity and lifetime number of matings. We demonstrate for the first time that males ejaculated significantly more eupyrenes and apyrenes in their lifetime after their young were exposed to juvenile rivals. Adult moths continued to produce eupyrene sperm, contradicting the previous predictions for lepidopterans. The eupyrene : apyrene ratio in the lifetime ejaculates remained unchanged in all treatments, suggesting that the sperm ratio is critical for reproductive success. Male juvenile exposure to other juveniles regardless of sex ratio caused significantly shorter adult longevity and faster decline in sperm ejaculation over successive matings. However, males from all treatments achieved similar number of matings in their lifetime. This study provides insight into adaptive resource allocation by males in response to juvenile sociosexual environment.

## Graphical Abstract

Using a polygamous moth, *Ephestia kuehniella*, we investigate how juvenile socio‐sexual environment influences lifetime sperm expenditure, mating frequency and survival in adult males. We demonstrate that after their immature stages are exposed to conspecific rivals, adults ejaculate more eupyrenes and apyrenes and continue to produce eupyrene sperm in their lifetime. Juvenile male exposure to conspecifics regardless of sex ratio shortens adult longevity and causes faster decline in sperm ejaculation over successive matings. Immature exposure does not change the eupyrene: apyrene ratio in the lifetime ejaculates and lifetime number of matings.

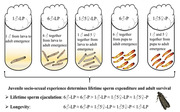

## Introduction

Animals are expected to adjust their behavior and physiology to gain a competitive edge in different sociosexual environments (Kasumovic & Brooks, 2011; Acasuso‐Rivero *et al*., 2019; Westneat *et al*., 2019). Various studies show that after adult males detect sperm competition risk, they raise their sperm expenditure for a higher paternity share (e.g., Parker, 1970; Gage, 1991; Simmons *et al*., 2007; Jarrige *et al*., 2015; Esfandi *et al*., 2020; Liu *et al*., 2020). To date, only a few studies have explored how male insects tailor their investment in sperm as a response to juvenile sociosexual settings. For example, adults from juveniles exposed to higher density of conspecific juveniles regardless of sex ratio ejaculate more sperm in their first mating (Gage, 1995; He & Miyata, 1997; Yamane & Miyatake, 2005; McNamara *et al*., 2010) or have higher sperm counts at emergence (Liu *et al*., 2021, 2022). Allen *et al*. (2011) report that after male juveniles are reared together, their adults transfer more sperm during the first mating. Yet, it is still unknown whether and how juvenile sociosexual environment influences lifetime sperm production and allocation in any animal species. It is also unclear whether exposure to similar sociosexual settings by different juvenile stages results in diverse lifetime sperm expenditure in adults.

Most lepidopteran species produce two distinct spermatozoa, the nucleate eupyrenes (fertile) and anucleate apyrenes (unfertile), and the most widely accepted notion is that spermatogenesis occurs in juvenile stages and eupyrene sperm production ends before adult emergence (Swallow & Wilkinson, 2002; Friedländer *et al*., 2005). However, it remains unclear whether juvenile sociosexual situations affect spermatogenesis during the adult stage. Although the apyrene sperm cannot fertilize eggs, they assist eupyrene in migration from female bursa copulatrix to spermatheca (Sakai *et al*., 2019; Chen *et al*., 2020; Konagaya *et al*., 2020; Hague *et al*., 2021), protect eupyrene sperm against a hostile female reproductive tract (Holman & Snook, 2008), and help win sperm competition games (Cook & Wedell, 1999; Wedell *et al*., 2009; Mongue *et al*., 2019). Therefore, the eupyrene : apyrene ratio could be essential for reproductive success in males. Though, it is unknown whether the juvenile sociosexual environment affects the sperm ratio in ejaculates transferred during the adult lifespan.

The number of sperm ejaculated by males should decrease over successive matings due to limited resources and aging (Wedell & Cook, 1999; Velde *et al*., 2011; Esfandi *et al*., 2015, 2020; Liu *et al*., 2020). However, it is not clear whether different juvenile experience alters lifetime ejaculation pattern. Because sperm production (Dewsbury, 1982; Van Voorhies, 1992; Olsson *et al*., 1997; Pitnick *et al*., 2006; Hayward & Gillooly, 2011; Lemaître *et al*., 2020) and matings (Martin & Hosken, 2004; McNamara *et al*., 2008; Oliver & Cordero, 2009; Metzler *et al*., 2016; Mautz *et al*., 2019; Jehan *et al*., 2020) are costly, males may not be able to maintain maximal reproduction and longevity simultaneously (Kirkwood, 1977; Roff, 2002). Nevertheless, many studies show that the adult sociosexual environment alters males’ reproductive investment but not their longevity (e.g., Janowitz & Fischer, 2010; Moatt *et al*., 2013; Esfandi *et al*., 2015; Leech *et al*., 2019). To date, knowledge of how juvenile sociosexual settings affect adult mating frequency and longevity is still lacking.

We used a polygamous moth, *Ephestia kuehniella*, to investigate how juvenile sociosexual environment influences lifetime sperm expenditure, mating frequency and survival in adult males. Under our experimental conditions, larval and pupal stages last about 29 and 8 days, respectively. Adults do not feed, and all resources are acquired during the larval stage. Adults become sexually mature at emergence and start mating at the onset of the first scotophase (Xu *et al*., 2008). Adult males increase their sperm allocation after exposure to rival cues during early adulthood (Esfandi *et al*., 2020; Liu *et al*., 2020). Adults have more sperm of both types at emergence after their pupae are exposed to higher density of conspecifics (Liu *et al*., 2021) but if such exposure starts at the larval stage, only eupyrene sperm increases at emergence (Liu *et al*., 2022), suggesting that larvae and pupae may respond to the same social context differently.

In the current study, we prepared thousands of larvae and manipulated larval–pupal and pupal density with varied sex ratios. Upon male adult emergence, we paired each male with a virgin female per day until his death. We dissected each mated female to count eupyrene and apyrene sperm transferred per mating and recorded males’ lifetime mating frequency and longevity. This is the first study on how juvenile sociosexual experience affects male adult longevity, and lifetime sperm production and allocation, eupyrene : apyrene sperm ratio and mating frequency in an insect. Knowledge presented here provides novel understanding of adaptive resource allocation by males in response to juvenile sociosexual environment.

## Materials and methods

### Insects and environmental conditions

We collected *E*. *kuehniella* larvae from a poultry farm, in Foxton, New Zealand and reared them to adults with their original food (a mixture of wheat and maize flour) in 20 transparent plastic cylinders (10.0 cm height × 8.0 cm diameter). We randomly selected about 300 newly emerged adults (ca. 1 : 1 sex ratio) from all cylinders and introduced them into a transparent plastic cage (24.0 cm height × 28.0 cm length × 28.0 cm width) with a porous plastic sheet at the bottom for oviposition. We collected eggs by pulling out the sheet and replacing it with a new one once every day for 10 days and incubated the eggs in Petri dishes (1.5 cm height × 8.5 cm diameter). We then inoculated 200 neonate larvae to 50 g standard diet (ad libitum) (21.75 g whole meal wheat flour, 21.75 g maize meal, 5 g glycerine, and 1.5 g yeast) in a plastic cylinder as mentioned above. We constantly maintained 20 such cylinders as the breeding colony for experimental insects.

We randomly transferred 300 newly emerged adults (ca. 1 : 1 sex ratio) from the colony to an aforementioned plastic cage for mating and subsequently letting females lay eggs in the cage. We then randomly collected 1000 neonate larvae from the cage to establish an experimental line. We reared these larvae individually in 2‐mL Eppendorf tubes, each of which held 0.25 g standard diet for food and pinholes in the lid for ventilation. To prepare virgin females for mating with focal males over the course of the experiment, we randomly collected about 1000 female pupae from the breeding colony and individually housed them in the Eppendorf tubes until use for experiment. We maintained the colony and experimental insects and carried out all experiments at 25 ± 1 °C and 60% ± 10% RH under the photoperiod of 10 : 14 h (dark : light).

### Pre‐adult socio‐sexual settings for focal males

**Fig. 1:**
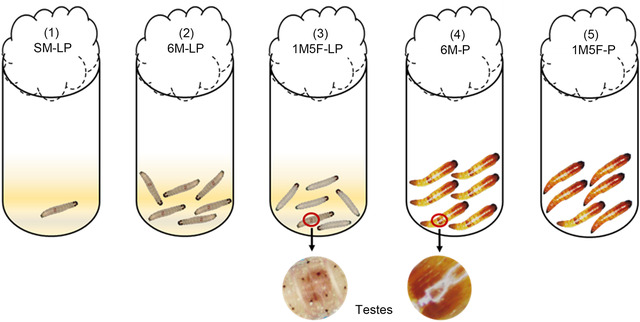
Socio‐sexual environment treatments for *E*. *kuehniella* males before eclosion: (1) SM‐LP, single male from the fourth instar larva to adult emergence; (2) 6M‐LP, six males together from the fourth instar larvae to adult emergence; (3) 1M5F‐LP, one male and five females together from the fourth instar larvae to adult emergence; (4) 6M‐P, six male pupae together for the entire pupal stage until adult emergence; and (5) 1M5F‐P, one male and five females together for the entire pupal stage until adult emergence.

The sex of the fourth instar larvae and pupae can be determined by visible testes in males’ abdomens (Fig. 1; Liu *et al*., 2021). We randomly selected newly molted fourth‐instar larvae (L) and newly pupated pupae (P) from the experimental line and transferred them into glass vials (7.5 cm height × 2.0 cm diameter) to create five sociosexual enviroments for focal males (M) (Fig. 1): (1) single male (SM‐LP)—one male was kept in a glass vial with a 0.25 g standard diet from the fourth instar larva to adult emergence; (2) six males (6M‐LP)—six males were raised in a glass vial with a 1.5 g standard diet from the fourth instar larvae to adult emergence; (3) one male and five females (1M5F‐LP)—one male and five females (F) were reared in a glass vial with a 1.5 g standard diet from the fourth instar larvae to adult emergence; (4) six male pupae (6M‐P)—six male pupae were maintained in a glass vial for the entire pupal stage until adult emergence, and (5) one male and five female pupae (1M5F‐P)—one male and five females were put in a glass vial for the entire pupal stage until adult emergence. All glass vials were covered with wool cotton at the top.

For all treatments, we monitored adult emergence hourly when the pupae turned dark brown (ca. 1 day before adult emergence). Immediately after males’ eclosion, we individually transferred them into clean glass vials and clearly labeled all vials. To keep conditions consistent, we only used males from vials where all individuals emerged for data collection. We considered the male from each SM‐LP, 1M5F‐LP and 1M5F‐P vial and all six males from each 6M‐LP and 6M‐P vial as focal males.

### Data collection

At the onset of the first scotophase following eclosion, we individually paired the focal males with 1‐day‐old virgin females randomly selected from the breeding colony, in transparent plastic cylinders (17.0 cm length × 6.5 cm diameter). Ten red light tubes (Sylvania, F36W/Red, Holland) 1.5 m above the cylinders were used for illumination. Because a male requires 24‐h recovery time to produce a full spermatophore again after each mating (Xu & Wang, 2009), we randomly assigned another 1‐day‐old virgin female to the focal male in the cylinder at the onset of the next scotophase. This procedure was repeated until the death of the focal male. We monitored each mating pair once every 15 min until mating ended and immediately removed the mated female from the cylinder. We recorded mating frequency (lifetime number of matings) and longevity (duration between emergence and death) of each focal male. We considered each focal male as a replicate. In total, we achieved 24, 23, 24, 22, and 24 replicates for treatments SM‐LP, 6M‐LP, 1M5F‐LP, 6M‐P, and 1M5F‐P, respectively.

To record males’ lifetime sperm allocation, we counted the number of sperm ejaculated in each mating via dissecting all mated females from the above experiment and extracting the spermatophores out from their bursa copulatrix. In total we dissected 123, 130, 133, 136, and 142 females for SM‐LP, 6M‐LP, 1M5F‐LP, 6M‐P, and 1M5F‐P, respectively. We placed the bursa copulatrix into a droplet of Belar saline solution on a cavity slide. Using two fine needles, we ruptured the spermatophore to release sperm under a stereomicroscope (Leica MZ12, Wetzlar, Germany). We then counted the number of bundles of eupyrene sperm under a phase‐contrast microscope (Olympus BX51, Tokyo, Japan). We calculated the total number of eupyrene sperm as the total number of bundles multiplied by 256, the number of eupyrene sperm per bundle (Garbini & Imberski, 1977). Afterward, the sample was thoroughly washed off the cavity slide and diluted in a glass vial with 30‐mL distilled water. We gently rotated the vial for about 30 s to deliver even dispersal of apyrenes in the vial. We took eight 10‐μL subsamples from the vial using a Gilson autopipette and placed them separately on a microscope slide. We counted the number of apyrene sperm of all eight subsamples under the phase‐contrast microscope and calculated the mean number per 10 μL as the sum of apyrene sperm in eight subsamples divided by eight. We then calculated the total number of apyrene sperm for each mating as the mean number of apyrenes per 10 μL multiplied by the dilution factor (3000) (Koudelová & Cook, 2001). The lifetime number of eupyrene and apyrene sperm ejaculated by a male adult is the sum of the sperm ejaculated in each mating.

### Statistical analysis

We analyzed all data using SAS 9.13 (SAS Institute Inc, USA) with a rejection level set at *P* < 0.05. Because the experimental design was pseudoreplicated, we analyzed the mating frequency and lifetime number of eupyrene and apyrene sperm transferred by male adults using a linear mixed‐effects model (MIXED procedure) (Millar & Anderson, 2004; Harrison *et al*., 2018), with treatment as a fixed factor and replicate nested into the vial (male source) as a random factor (Davies & Gray, 2015; Harrison *et al*., 2018). We applied a Tukey's test in the model for multiple comparisons between treatments. A log‐rank test (LIFETEST procedure) was applied to compare the survival probability of focal males between treatments. The relationship between the total number of eupyrene and apyrene ejaculated was analyzed by a general linear model (GLM procedure) and an analysis of covariance (ANCOVA) was used to compare the slopes of regression lines between treatments (Liu *et al*., 2021). We used a linear mixed‐effects model with repeated measures (MIXED procedure) to test how treatment affected males’ sperm allocation in successive matings. We set treatment, mating frequency and their interaction as the fixed effects in the model with a subject effect of focal male in the statement of “REPEATED / TYPE = cs SUBJECT = focal male” after the model. A CONTRAST statement was then applied to compare the slopes of regression lines of sperm ejaculation over successive matings between treatments. Because the moths used in the current study were from the same batch reared under the same conditions as in Liu *et al*. (2021, 2022), we used a two‐sample t test (TTEST procedure) to compare the number of eupyrene and apyrene sperm and their ratio in lifetime ejaculates with those recorded at emergence (Liu *et al*., 2021, 2022).

## Results

### Effect of socio‐sexual environment during juvenile stages on lifetime sperm allocation

**Fig. 2:**
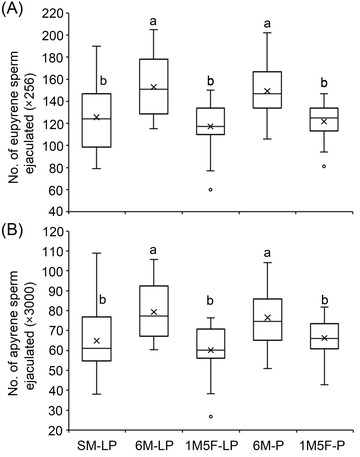
Effects of preadult socio‐sexual environment on lifetime eupyrenes (A) and apyrenes (B) ejaculated by *E*. *kuehniella* males. SM‐LP, single male from fourth instar larva to adult emergence; 6M‐LP, six males together from fourth instar larvae to adult emergence; 1M5F‐LP, one male and five females together from fourth instar larvae to adult emergence; 6M‐P, six male pupae together for the entire pupal stage until adult emergence; and 1M5F‐P, one male and five females together for the entire pupal stage until adult emergence. For each box plot, the lower and upper box lines indicate 25% and 75% of scores falling beyond the lower and upper quartiles, respectively; the line and “×” show the median score and means, respectively; the “⊥” and “T” are the lower and upper whiskers representing scores outside the 50% middle; the circles are the outliers of minimum or maximum scores. Boxes with different letters denote significant differences between treatments (*P* < 0.05).

**Fig. 3:**
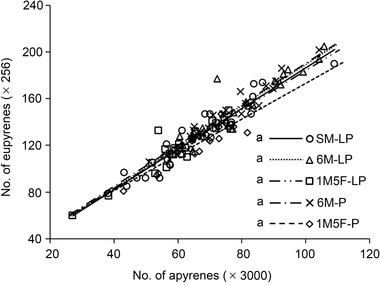
Relationship between the number of eupyrene and apyrene sperm ejaculated in *E*. *kuehniella* males’ lifetime. For SM‐LP, eupyrene = 12.31 + 1.74 × apyrene (*F*_1,22_ = 204.10, *P* < 0.001); for 6M‐LP, eupyrene = 13.17 + 1.76 × apyrene (*F*_1,20_ = 130.51, *P* < 0.001); for 1M5F‐LP, eupyrene = 16.13 + 1.68 × apyrene (*F*_1,21_ = 126.29, *P* < 0.001); for 6M‐P, eupyrene = 14.86 + 1.76 × apyrene (*F*_1,20_ = 275.94, *P* < 0.001); and for 1M5F‐P, eupyrene = 20.07 + 1.53 × apyrene (*F*_1,22_ = 103.42, *P* < 0.001).

Males that were exposed to conspecific males during larval–pupal stages (6M‐LP) or the pupal stage (6M‐P) ejaculated significantly more eupyrene and apyrene sperm than those that were exposed to conspecific females during larval–pupal stages (1M5F‐LP) or the pupal stage (1M5F‐P) or reared singly during larval–pupal stages (SM‐LP) (*F*_4,48_ = 4.11, *P* = 0.006 for eupyrene; *F*_4,48_ = 4.58, *P* = 0.003 for apyrene) (Fig. 2). The lifetime number of eupyrenes and apyrenes ejaculated was significantly positively correlated in all treatments (*P* < 0.001), with no significant difference in the slopes of regression lines between treatments (*F*_4,105_ = 0.32, *P* = 0.864) (Fig. 3).

**Fig. 4:**
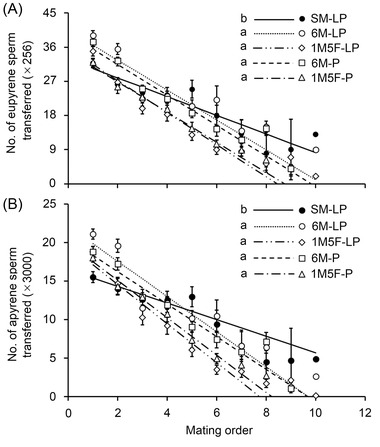
Effects of preadult social environment on eupyrenes (A) and apyrenes (B) ejaculated by *E*. *kuehniella* males in relation to mating order (MO). Eupyrene: for SM‐LP, sperm = 32.84 – 2.46 × MO (*F*_1,98_ = 56.27, *P* < 0.001); for 6M‐LP, sperm = 40.51 – 3.95 × MO (*F*_1,108_ = 201.38, *P* < 0.001); for 1M5F‐LP, sperm = 35.67 – 4.23 × MO (*F*_1,110_ = 251.93, *P* < 0.001); for 6M‐P, sperm = 39.49 – 4.02 × MO (*F*_1,113_ = 218.17, *P* < 0.001); and for 1M5F‐P, sperm = 34.89 – 4.00 × MO (*F*_1,117_ = 194.98, *P* < 0.001). Apyrene: for SM‐LP, sperm = 16.48 – 1.08 × MO (*F*_1,98_ = 33.38, *P* < 0.001); for 6M‐LP, sperm = 22.11 – 2.28 × MO (*F*_1,108_ = 157.73, *P* < 0.001); for 1M5F‐LP, sperm = 19.62 – 2.54 × MO (*F*_1,110_ = 237.20, *P* < 0.001); for 6M‐P, sperm = 20.45 – 2.11 × MO (*F*_1,113_ = 194.50, *P* < 0.001); and for 1M5F‐P, sperm = 19.91 – 2.42 × MO (*F*_1,117_ = 186.22, *P* < 0.001). Points and vertical lines represent means and standard errors, respectively.

Both eupyrene (Fig. 4A) and apyrene (Fig. 4B) sperm ejaculated declined significantly over successive matings (*P* < 0.001). However, both types of sperm ejaculated declined significantly faster in males exposed to conspecifics (6M‐LP, 1M5F‐LP, 6M‐P, and 1M5F‐P) than in ones unexposed to conspecifics (SM‐LP) (*F*_4,635_ = 4.73, *P* < 0.001 for eupyrene; *F*_4,635_ = 7.96, *P* < 0.001 for apyrene) and there was no significant difference in slopes among exposed males (eupyrene: *F*_3, 514_ = 0.63, *P* > 0.05; apyrene: *F*_3, 514_ = 0.96, *P* > 0.05) (Fig. 4).

### Comparison between the number of sperm ejaculated in lifetime and that counted at emergence

In treatments 6M‐LP, 6M‐P, and SM‐LP, the lifetime number of eupyrene sperm ejaculated (Fig. 2A) was significantly higher than that counted at emergence (*t*_50_ = 4.20, *P* < 0.001 for 6M‐LP; *t*_50_ = 2.52, *P* = 0.016 for 6M‐P; *t*_54_ = 3.09, *P* = 0.004 for SM‐LP) (Liu *et al*., 2021, 2022).The lifetime number of apyrene sperm ejaculated (Fig. 2B) was also significantly higher than that measured at emergence in all five treatments (*t*_50_ = 8.61, *P* < 0.001 for 6M‐LP; *t*_51_ = 3.33, *P* = 0.002 for 1M5F‐LP; *t*_50_ = 5.58, *P* < 0.001 for 6M‐P; *t*_52_ = 3.21, *P* = 0.002 for 1M5F‐P; *t*_54_ = 5.15, *P* < 0.001 for SM‐LP) (Liu *et al*., 2021, 2022). Furthermore, the apyrene : eupyrene ratio in lifetime ejaculates (6 : 1) (Fig. 2 ) was significantly higher than that (5　:　1) at emergence (Liu *et al*., 2021, 2022) (*t*_50_ = 7.74, *P* < 0.001 for 6M‐LP; *t*_51_ = 5.99, *P* < 0.001 for 1M5F‐LP; *t*_50_ = 6.03, *P* < 0.001 for 6M‐P; *t*_52_ = 6.54, *P* < 0.001 for 1M5F‐P; *t*_54_ = 3.80, *P* = 0.001 for SM‐LP).

### Effect of socio‐sexual environment during juvenile stages on mating frequency and longevity

**Fig. 5:**
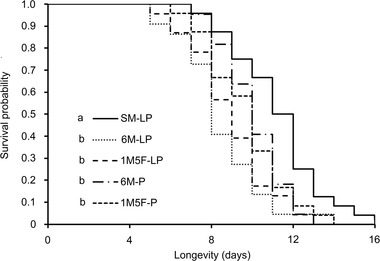
Effects of preadult socio‐sexual environment on adult male longevity in *E*. *kuehniella*. SM‐LP, single male from fourth instar larva to adult emergence; 6M‐LP, six males together from fourth instar larvae to adult emergence; 1M5F‐LP, one male and five females together from fourth instar larvae to adult emergence; 6M‐P, six male pupae together for the entire pupal stage until adult emergence; and 1M5F‐P, one male and five females together for the entire pupal stage until adult emergence. Lines with different letters are significantly different (*P* < 0.05).

Preadult sociosexual exposure had no significant effect on the number of matings adult males achieved in their lifetime (mean ± *SE* = 5.13 ± 0.39, 5.91 ± 0.43, 5.78 ± 0.37, 6.18 ± 0.37, and 5.92 ± 0.30 for SM‐LP, 6M‐LP, 1M5F‐LP, 6M‐P, and 1M5F‐P, respectively) (*F*_4,48_ = 1.38, *P* = 0.255). However, regardless of sex ratio, adult males that were exposed to conspecific individuals during larval–pupal stages or the pupal stage (6M‐LP, 1M5F‐LP, 6M‐P, and 1M5F‐P) lived significantly shorter than those that were reared singly (SM‐LP) (*x* ^2^_4_ = 21.44, *P* < 0.001), and all exposed males had similar longevity (*P* > 0.05) (Fig. 5).

## Discussion

The present study shows that both juvenile stages of *E*. *kuehniella*, larvae and pupae, are sensitive to their sociosexual environment and their experience affects lifetime sperm production and allocation and adult longevity but not eupyrene : apyrene ratio and mating frequency. We demonstrate for the first time that adult males developing from juveniles reared with juvenile rivals transferred significantly more eupyrenes (Fig. 2A) and apyrenes (Fig. 2B) in their lifetime than those from juveniles raised solitarily or with juvenile mates. Our findings provide strong evidence that the impact of sociosexual environment during juvenile stages continues throughout the adult stage. Furthermore, the sperm allocation patterns remained the same following exposure either from late instar larval to pupal stages or just during the pupal stage (Fig. 2). This suggests that the late juvenile stage is a critical period for building up the long‐term memory of the preadult social environment in insects.

Using the same batch of moths reared under the same conditions as in Liu *et al*. (2021, 2022), we show that the number of sperm ejaculated in lifetime (Fig. 2) was significantly higher than that counted at emergence (Liu *et al*., 2021, 2022) in *E*. *kuehniella*. These findings suggest that the production of both eupyrene and apyrene sperm continues during the adult stage in lepidopterans, contradicting previous perceptions (Swallow & Wilkinson, 2002; Friedländer *et al*., 2005). The sperm ratio (apyrene : eupyrene) in lifetime ejaculates (Fig. 2) was also significantly higher than that at emergence (Liu *et al*., 2021, 2022), supporting previous findings that apyrenes are cheaper to produce than eupyrenes (Silberglied *et al*., 1984; Cook & Gage, 1995). Our study reveals that the sperm ratio in lifetime ejaculates remained the same regardless of treatments during juvenile stages (Fig. 3), suggesting that the sperm ratio in ejaculates is critical for reproductive success.

Similar to previous findings (e.g., Wedell & Cook, 1999; Velde *et al*., 2011; Esfandi *et al*., 2020; Liu *et al*., 2020), we show that the number of sperm ejaculated by males significantly decreased over successive matings (Fig. 4). These patterns fit the general prediction that males suffer from reduced quantity of their sperm with age (Dewsbury, 1982; Fricke & Maklakov, 2007; Vega‐Trejo *et al*., 2019). However, the ejaculation of both eupyrene and apyrene sperm declined significantly faster over time in males whose juveniles were exposed to conspecific juveniles of any sex ratio than in those unexposed (Fig. 4). Higher sperm production before emergence in the exposed males (Liu *et al*., 2021, 2022) may exacerbate sperm senescence (Ball & Parker, 1996; Reinhardt, 2007; Pizzari *et al*., 2008) so that they are of greater urgency to expel the accumulated aged sperm in their reservoirs to gain reproductive fitness. This may result in ejaculation of more sperm in their first couple of matings, raising the starting points of the linear lines and leading to steeper slopes (Fig. 4).

Adult *E*. *kuehniella* males had significantly shorter longevity after their juveniles were exposed to conspecific juveniles of any sex ratio as compared to those whose young were individually reared (Fig. 5). Because most spermatogenesis occurs during juvenile stages (Friedländer *et al*., 2005; Liu *et al*., 2021, 2022) and sperm production entails significant costs (Dewsbury, 1982; Van Voorhies, 1992; Olsson *et al*., 1997; Pitnick *et al*., 2006; Hayward & Gillooly, 2011; Lemaître *et al*., 2020), we suggest that the increase of resource allocation to sperm production in the presence of conspecifics during juvenile stages (Liu *et al*., 2021, 2022) causes the early death of male adults. We show that males in different treatments achieved the same number of matings in their lifetime, suggesting that the number of matings is ultimately important for maximal reproductive fitness regardless of juvenile experience in *E*. *kuehniella* males.

In conclusion, the present study provides the first evidence that adult *E*. *kuehniella* males ejaculate significantly more eupyrene and apyrene sperm in their lifetime after exposure to rivals during the larval–pupal or pupal stage. In contrary to previous predictions for lepidopterans, we show that adults continue to produce sperm of both types. Despite different lifetime sperm allocations among treatments, the apyrene : eupyrene ratio remains 6 : 1, implying that the sperm ratio in ejaculates is critical for reproductive success. While both types of sperm ejaculated decrease over successive matings in all treatments, the rate of decrease is faster in males exposed to conspecifics during juvenile stages. This may result from the fact that the exposed males produce more sperm before emergence and ejaculate more in their first mating. Adults from juveniles exposed to conspecific juveniles of any sex ratio have shorter longevity probably because exposed juveniles allocate more resources to sperm production and trade off adult survival. Finally, all *E*. *kuehniella* males have similar number of matings in their lifetime regardless of whether their juveniles are exposed to conspecific juveniles or not. The knowledge generated here provides insight into adaptive resource allocation by males in response to sociosexual experience of different juvenile stages.

## Disclosure

We declare we have no conflict of interest associated with this publication.
